# Determining Out-of-Field Doses and Second Cancer Risk From Proton Therapy in Young Patients—An Overview

**DOI:** 10.3389/fonc.2022.892078

**Published:** 2022-05-31

**Authors:** Maite Romero-Expósito, Iuliana Toma-Dasu, Alexandru Dasu

**Affiliations:** ^1^The Skandion Clinic, Uppsala, Sweden; ^2^Oncology Pathology Department, Karolinska Institutet, Stockholm, Sweden; ^3^Medical Radiation Physics, Stockholm University, Stockholm, Sweden; ^4^Medical Radiation Sciences, Department of Immunology, Genetics and Pathology, Uppsala University, Uppsala, Sweden

**Keywords:** proton therapy, pediatric patient, out-of-field dose, second cancer risk, brain and other nervous system cancer

## Abstract

Proton therapy has the potential to provide survival and tumor control outcomes comparable and frequently superior to photon therapy. This has led to a significant concern in the medical physics community on the risk for the induction of second cancers in all patients and especially in younger patients, as they are considered more radiosensitive than adults and have an even longer expected lifetime after treatment. Thus, our purpose is to present an overview of the research carried out on the evaluation of out-of-field doses linked to second cancer induction and the prediction of this risk. Most investigations consisted of Monte Carlo simulations in passive beam facilities for clinical scenarios. These works established that equivalent doses in organs could be up to 200 mSv or 900 mSv for a brain or a craniospinal treatment, respectively. The major contribution to this dose comes from the secondary neutrons produced in the beam line elements. Few works focused on scanned-beam facilities, but available data show that, for these facilities, equivalent doses could be between 2 and 50 times lower. Patient age is a relevant factor in the dose level, especially for younger patients (by means of the size of the body) and, in addition, in the predicted risk by models (due to the age dependence of the radiosensitivity). For risks, the sex of the patient also plays an important role, as female patients show higher sensitivity to radiation. Thus, predicted risks of craniospinal irradiation can range from 8% for a 15-year-old male patient to 58% for a 2-year-old female patient, using a risk model from a radiological protection field. These values must be taken with caution due to uncertainties in risk models, and then dosimetric evaluation of stray radiation becomes mandatory in order to complement epidemiological studies and be able to model appropriate dose–response functions for this dose range. In this sense, analytical models represent a useful tool and some models have been implemented to be used for young patients. Research carried out so far confirmed that proton beam therapy reduces the out-of-field doses and second cancer risk. However, further investigations may be required in scanned-beam delivery systems.

## Introduction

Concern about second cancer in patients who survived a primary malignancy has increased steadily over the past decades. Multiple epidemiological studies have focused on evaluating the risk of these second malignancies (1). The absolute risk of radiation-induced second cancer rates has been estimated to be in the order of 1% (2). Better estimates of this risk are made by studies with longer follow-up, which, in turn, implies that patients were treated with older technologies different from the current highly conformal therapies. Therefore, there is an intrinsic uncertainty about the actual risk. However, although risks were low, the high and growing number of patients affected must be considered. While the World Health Organization predicted in 2003 approximately 15 million new cancer patients by the year 2020 (3), the final value was 19.3 million (4). In addition, the Surveillance, Epidemiology, and End Results (SEER) Program registries show a 5-year relative survival of 67.2% (5). All these patients will be exposed to radiation from imaging procedures on a routine basis (6), and at least 50% will receive a radiotherapy (RT) treatment. Thus, second cancer risks should be considered although the benefit of the treatment is clearly confirmed.

From the point of view of medical physicists, the main contribution to this topic is to perform accurate estimates of the doses received by the patient. Determination of the dose delivered to the treatment target and closest organs [the so-called organs at risks (OARs)] is rather accurately carried out during planning by the treatment planning system (TPS). However, TPS can be trusted for doses above 5% of the prescription dose (7). Below this level and for the rest of the patient, the out-of-field dose is defined and has been the focus of many studies concerned with the second cancer induction.

Out-of-field dose or stray dose is due to all secondary particles produced by the interaction of the treatment beam with the elements of the delivery system and the patient. In the case of photon RT, the secondary particles are photons and neutrons (the latter when the linac operates at high energy, > 10 MV). In the case of particle therapy, in addition to photons and neutrons, other nuclear fragments can be produced in interactions. The contribution of each type of particle is different among the techniques. Xu et al. (1) published a comprehensive review on studies of out-of-field doses in external-beam radiation treatments, including both photon and particle therapies. By 2017, the American Association of Physicists in Medicine (AAPM) published a Task Group Report (No. 158) on the measurement and calculation of out-of-field doses (8). This code of practice, apart from updating the studies, discusses both the uses of dosimeters and phantoms in experimental evaluations and the calculation techniques. Special attention was paid on how to report doses, and some recommendations for practice were included as well. More recent overviews can be found in Mazonakis and Damilakis (9), which focused on photon RT, and in Hägl and Schneider (10), describing the state of art in the evaluation of neutron stray doses in proton beam therapy (PBT).

Out-of-field doses and second cancers lead to a greater concern in the case of young patients (≤21 years of age). First, children are more radiosensitive than adults (11), and secondly, after a successful treatment, their expected lifetime will be longer. Consequently, these factors are likely to lead to a higher risk of secondary cancer. However, this risk has to be regarded from the perspective of the treatment objective and should not prevent a patient from receiving RT.

The physical properties of PBT provide superior dose distributions compared to photons; this fact is especially relevant in pediatric RT (12). From 2004 to 2012, the proportion of children receiving PBT was significantly increasing over time from <1% to 15% (13). In USA, the National Cancer Institute estimated, by mid-2021, 10,500 new cases of cancer among children from birth to 14 years for the whole year (14). Cancer incidence rates reported by the Childhood Cancer Data Initiative were 196 and 185 per 1,000,000 for male and female patients, respectively (15). Among all the cancer sites, the most common after leukemia are cancers of the brain and the central nervous system (CNS). These cancers represent 16.4% of all new childhood cancer cases with a 5-year relative survival of 74.9%. PBT for the treatment of pediatric cancers of the CNS has been found to provide survival and tumor control outcomes comparable and frequently superior to photon therapy. Furthermore, the use of protons was shown to decrease the incidence of severe acute and late toxicities, including reduced severity of endocrine, neurological, cognitive, and quality-of-life deficits (16). At many facilities, pediatric patients represent a substantial portion of those receiving proton treatment, and CNS tumors comprise a large proportion of this group (13). An international survey carried out to evaluate the patterns of PBT in 2016 showed that 48% of pediatric patients (from a total 1,860 patients) were treated for CNS tumors, with medulloblastoma, ependymoma, low-grade glioma, and craniopharyngioma being the most frequent tumor types (17). An additional 14% of patients were treated for other head and neck tumors. Overall, 34% of patients were treated with passive scattering, 15% with uniform scanning beam, and 51% with pencil beam scanning. Data from the Pediatric Proton Consortium Registry with a total of 1,854 children enrolled by September of 2017 showed that majority of children received curative craniospinal irradiation (CSI) (17%) or involved field RT (58%) using mainly passive scattering (68%) vs. pencil-beam scanning (32%) proton therapy (18). More children with non-CNS tumors received pencil-beam scanning (39%) compared with CNS (28%). A more recent epidemiological study showed that the vast majority of pediatric patients worldwide have been treated using a passive modulation proton technique to date (19). This single-institution retrospective study of pediatric patients treated with double-scattered proton therapy for benign and malignant solid tumors found a risk of developing a second solid tumor of 1.7% if irradiated at age ≤5 years versus 0.1% if older (*p* < 0.0005). One limitation of the study is related to the latency of second tumors. The median follow-up was 3.3 years, and some tumors present higher latencies. In a work from the Childhood Cancer Survivor Study (CCSS) where the median follow-up was of 22.7 years after diagnosis, the analysis showed a 30-year cumulative incidence of 7.9% (20). More significantly, as childhood cancer survivors progress through adulthood, the risk of subsequent neoplasms increases.

In this context, the purpose of this manuscript is to offer an overview of the determination of out-of-field doses and the prediction of second cancer risk so far for young patients receiving PBT. Publications were considered when published from 2008 onward [from the publication of the review of Xu et al. (1)].

It is worth pointing out the importance of including the exposures during imaging procedures for the comprehensive study of the patient doses in relation to the probability of second cancer induction (6). The recent work of Marcu et al. (21) offers a systematic review on epidemiological studies covering cumulative doses and cancer risks in children and young adults’ examinations.

## Secondary Particle Production and Delivery Technique

Secondary particles in proton therapy are produced in nuclear inelastic interactions between the projectile and target nuclei both in the beamline components and in the patient. The process can be described by the abrasion–ablation or cascade–evaporation model (22). In short, protons, neutrons, and light fragments are emitted (with energies that may exceed even a hundred MeV) mostly in the forward direction (proton beam direction) while the residual nucleus is left in an equilibrium state, with a certain excitation energy (23). The remaining nucleus follows a de-excitation process leading to lower-energy secondaries, emitted more or less isotropically. After this emission, the final excitation energy is released by γ-rays. Neutrons and photons, as long-range secondary particles, can affect tissues far away from the target. This component has been concisely referred to as the aura of dose distribution (24).

The contribution and the main sources of these secondary particles are directly related to the beam delivery system. To cover all the tumor volume, the narrow pristine Bragg peak must be extended to form the so-called spread-out-Bragg-peak (SOBP). This can be done either by passive modulation of the primary beam, or by scanning the tumor volume with a millimeter-wide beam magnetically deflected (22). In passive scattering PBT (PS-PBT) the proton beam goes through and interacts with several elements such as the range modulation wheel (RMW), scatter foils, collimators, range compensators, or patient-specific apertures before reaching the patient (25). All these elements become a source of secondary neutrons, often referred to as external neutrons or spray (24), that can reach the patient. Monte Carlo (MC) simulations have shown that most of the neutrons that do reach the patient are generated in the precollimators, and the patient-specific aperture (26). In the proton pencil beam scanning PBT (PBS-PBT) technique, magnets steer a small pencil beam of protons to specific positions within a tumor target without the need for apertures or compensators (27). Unless a range shifter (RS) is used at the nozzle exit, a negligible amount of material is in the beam path and, therefore, external neutrons are hardly produced (22). Additional to the external neutrons, there are nuclear interactions between the proton beam and the patient tissue. Neutrons generated in these reactions, the so-called internal neutrons, are unavoidable. In short, it is considered that external neutrons are the main contributors to stray dose in patients in PS-PBT, while internal neutrons are in PBS-PBT (28).

As previously mentioned, de-excitation γ-rays are also produced by the nuclear reactions. However, the high relative biological effectiveness (RBE) of neutron in comparison to photons is responsible for the major focus on neutrons.

## Out-of-Field Doses in Young Patients’ Treatments

The majority of studies dealing with stray radiation have been carried out by MC simulations. Some advantages of the simulations are the possibility to perform systematic studies showing the effects of changing several irradiation parameters and to evaluate separately internal and external neutron contribution in PS-PBT. As stated by ICRP, the equivalent dose in an organ is the recommended quantity for subsequent risk estimates for specific individuals (29). Then, several works reported their results using this quantity. However, some authors preferred to report absorbed dose to avoid increasing uncertainties due to the use of the radiation weighting factors (*w_R_*). Another relevant aspect is that when reporting results, absorbed or equivalent doses are normalized by the prescription dose, which can be expressed in terms of physical proton dose in Gy or in terms of therapeutic dose in Gy (RBE). Then, it is important to specify which one is used to enable comparisons, becoming mandatory if the comparison is with photon treatments. However, the review process showed that all works used Sv/Gy to report their results, although they referred to Sv/Gy (RBE) (usually in the text, expressions like “Equivalent dose per therapeutic dose” were included). Despite this, in the present work, the unit Sv/Gy (RBE) will be preferably used. Results were corrected by RBE = 1.1 for those works reporting results in terms of the physical dose. Hereafter, the following sections cover the overview of works focused on brain irradiation and craniospinal irradiation (CSI).

### Out-of-Field Doses in Brain Irradiations

Bonfrate et al. (30) performed the most comprehensive study on the influence of several treatment parameters on neutron production. The work modeled a passive double scattering beam line used for treating a 10-year-old female phantom in the brain. Each dependency study is summarized below.

First, the selection of beam incidence can modify the level of the doses. They compared an anterior–superior (SUP) incidence (patient oriented parallel to beam axis) and lateral (LAT) incidence (patient oriented perpendicular to beam axis). Neutron absorbed doses in thyroid and bladder were 123 µGy/Gy (RBE) and 22 µGy/Gy (RBE), respectively, in the SUP field, while for the LAT field, they were 321 µGy/Gy (RBE) and 76 µGy/Gy (RBE), respectively. On the one hand, these results showed that organ doses decrease as the distance to the target increases. On the other hand, lateral incidences produce higher doses as organs are at shorter distances from the patient collimator, which is the beam element with a major neutron contribution to organ doses [approximately 70% according to Matsumoto et al. (31)]. In addition, in the superior incidence, the head and neck and thoracic regions become a neutron shield for the rest of the body.

Neutron absorbed doses increase as proton energy and width modulation increase. For example, an averaged factor of 2.5 was found between absorbed doses when the proton energy changed from 162 to 219 MeV. The increase with wider modulation is a bit more complex because it presents a saturation effect. When modulation width was changed from 1 cm to 3 cm, doses increase by 38% on average, but from 3 to 5.6 cm, the change was almost negligible. This behavior was also seen in ambient dose equivalent measured in the treatment room (32). As modulation width increases, a larger proton fluence is needed to deliver the same dose to the target, but beyond certain widths, the required increase in fluence becomes too small to affect the neutron production rate. The relevant consequence of the energy and modulation effect is that larger treatment volumes that are deeper in the patient will cause significantly higher neutron equivalent doses (33).

Target volume is also related to the field size fixed by the collimator aperture. However, internal and external neutrons have opposite effects. Meanwhile, increasing the diameter of collimator leads to a lower production of external neutrons as less material blocks the proton beam, the production of internal neutron increases as more protons interact with the patient. Zacharatou-Jarlskog et al. (33) performed simulations distinguishing internal and external neutrons in PS-PBT. They found that while for small fields the contribution of external neutrons could be more than 99%, it could be reduced to 60% for larger fields. In addition, due to the distance effect previously mentioned, internal neutrons have a significant contribution for organs near the target volume and a low contribution for organs located far from it (30). The complex inter-relationships hamper the derivation of a general trend. In fact, Zacharatou-Jarlskog et al. (33) disagreed with simulations of Bonfrate et al. (30). While the former obtained equivalent doses lower with larger fields, the latter found higher absorbed doses for larger collimator aperture in the SUP field. Furthermore, in the LAT incidence, absorbed doses only increased in head and neck organs and decreased in thoracic and pelvic organs. The disagreement with Zacharatou-Jarlskog et al. (33), where lateral fields were considered, can be justified by the differences in the particular configuration of the beam line in the passive facilities modeled.

Collimator and compensator thickness changes had a similar impact on absorbed dose and the incidence dependence (30). For example, for the SUP field, neutron absorbed doses tend to decrease when increasing thickness, about 13% for a change from 5 to 8.5 cm. Again, for the LAT field, organ doses presented a similar reduction but only for organs between the target and the heart. The rest of the organs were almost insensitive to the variation of collimator thickness. Finally, increasing the air gap size from 1 cm to 12 cm led to neutron absorbed doses decreasing on average by 19%, 12%, and 5% for organs located in the head and neck, thoracic, and pelvic regions, respectively.

Certainly, the most relevant effect to study for young patients is the one related to the age of the patient or, in other words, the size of the patient. Both Zacharatou-Jarlskog et al. (33) and Sayah et al. (34) performed simulations of brain irradiation in PS-PBT in several hybrid phantoms covering representative ages for male and female patients. Phantoms used were those developed by the University of Florida (35). It is important to note that these models are being considered by ICRP to be used as pediatric reference computational phantoms as they take into account not only changes in the geometry as a function of age but also changes in the organ-specific material composition as a function of age (34). The general trend was that secondary neutron dose received by an organ decreases as the phantom’s age increases; this is a consequence of the reduction in dose as the organ is farther from the target volume. An older patient has a larger size and then distances between organs are also larger. Sayah et al. (34) evaluated brain treatment using 5 field incidences (including LAT, SUP, and oblique incidences) and using the same target volume. They also reported higher doses for lateral incidences, and the antero-superior incidence delivered the lowest doses. They found, for instance, that the neutron equivalent dose in salivary glands for 1-year phantom was 1.2 times higher than for adults. This factor reached a value of 2.7 and 3.2 for bladder and uterus/prostate. As expected, differences between the child and the adult increased as the organ was farther from the target. Sayah et al. (34) also evaluated the contribution of secondary photons for the 5-year-old phantom. Their results reported a contribution between 4% and 16% of the total equivalent dose. This low contribution justifies that most works focused on neutron contamination in PS-PBT.

**Figure 1:**
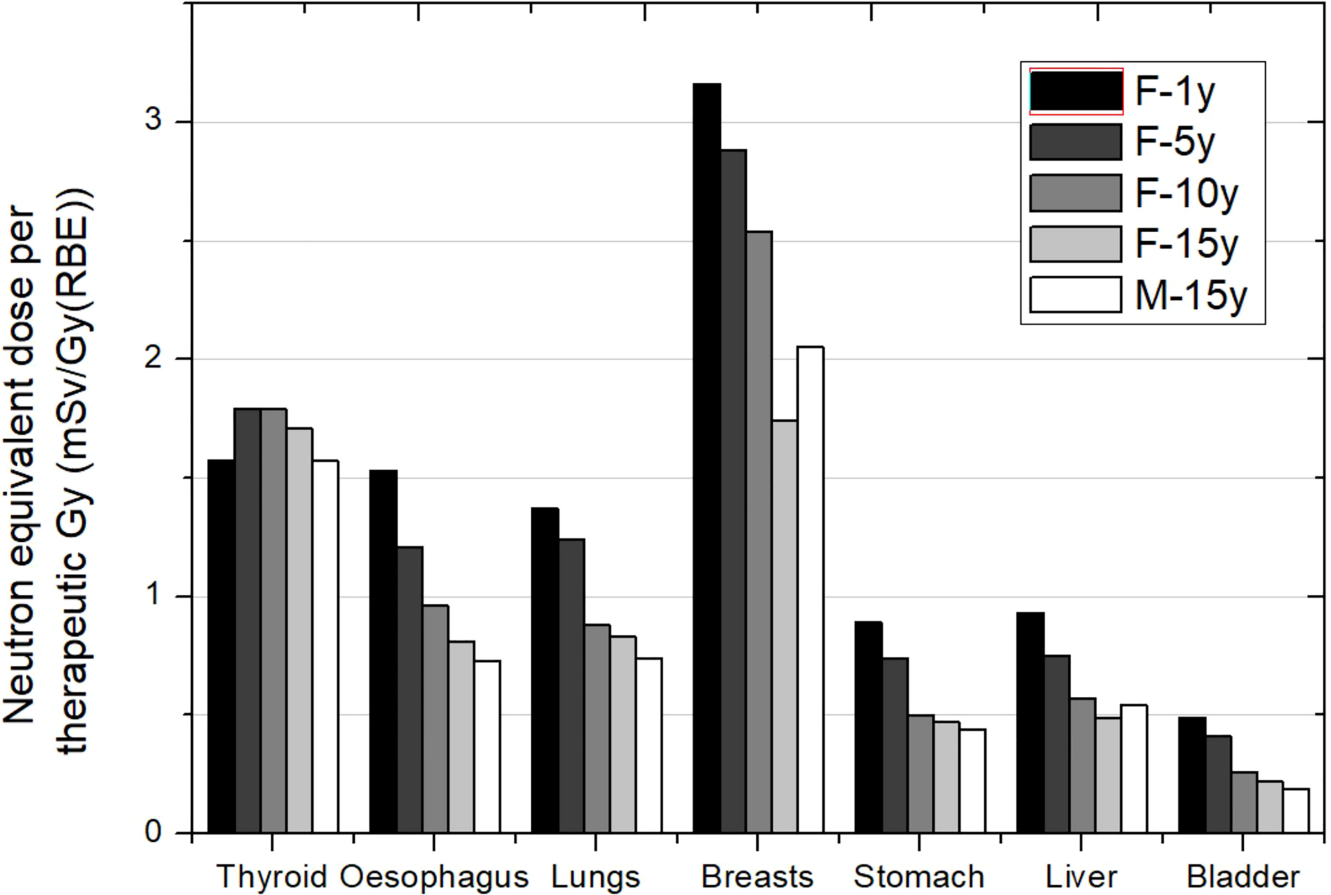
Neutron equivalent doses per therapeutic Gy in selected organs for a brain treatment in the same passive scattering proton beam facility and several patient ages. In the legend, the sex (F, female; M, male) and age of the patient (#y = number of years) are specified. Data extracted from Sayah et al. (34).

**Figure 2:**
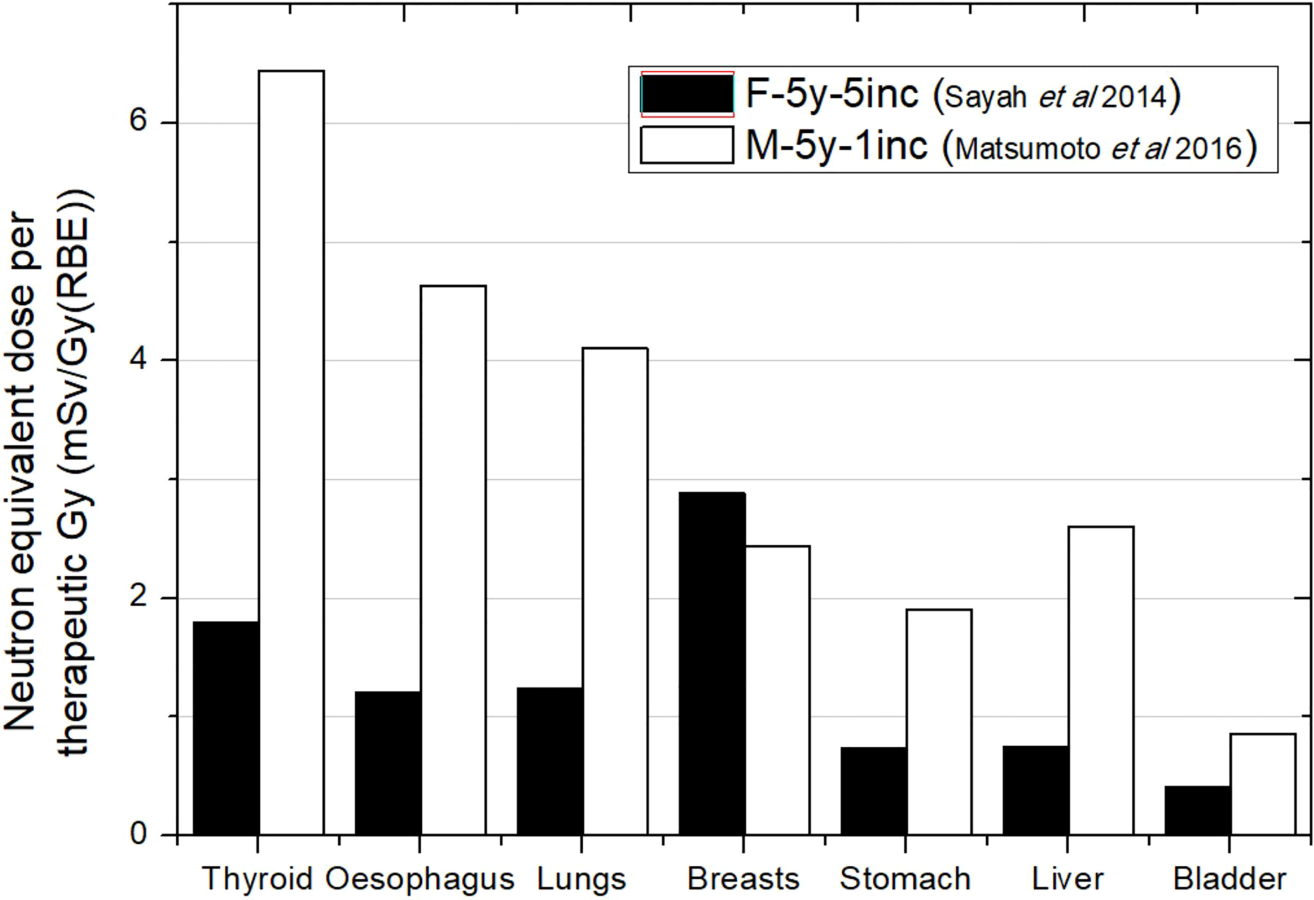
Neutron equivalent doses per therapeutic Gy in selected organs for a brain treatment in two different passive scattering proton beam facilities. Data extracted from Matsumoto et al. (31) and Sayah et al. (34) for the same age phantom (5 years) but different target volumes and number of incidences: 83 cm^3^ in Matsumoto et al. (31) and 92 cm^3^ in Sayah et al. (34). In the legend, the sex (F, female; M, male), age of the patient (#y = number of years), and number of incidences (#inc = number of incidences) are specified.

**Figure 3:**
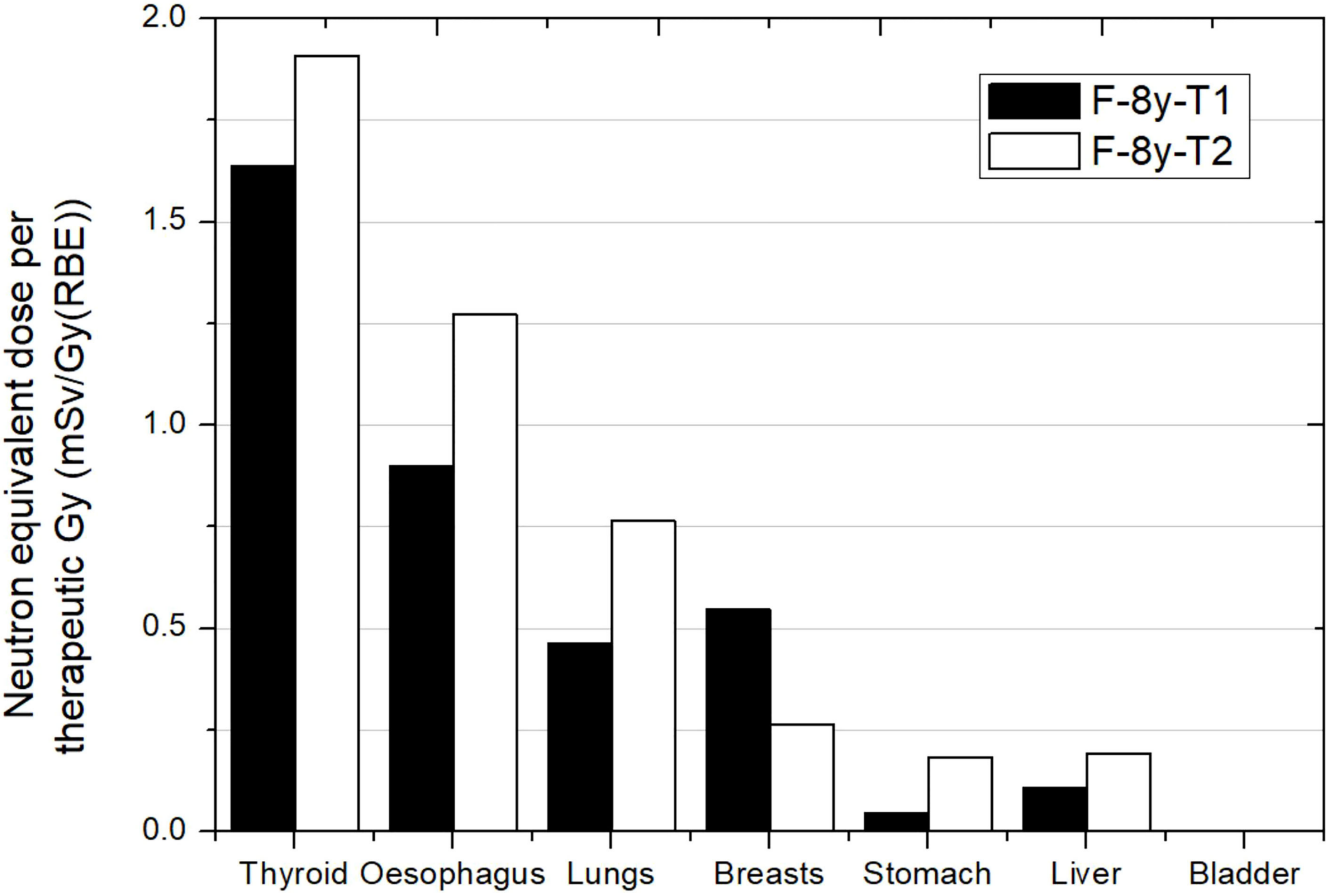
Neutron equivalent doses per therapeutic Gy in selected organs for 2 different brain treatments in the same passive scattering proton beam facility. T1 corresponds to an irradiation with a lateral incidence and a target volume of 514 cm^3^. T2 corresponds to an irradiation with an oblique-inferior incidence and a target volume of 306 cm^3^. Equivalent doses in bladder were too low for the plot scale. Data extracted from Zacharatou-Jarlskog et al. (33).

**Figures 1**–**3** depict the neutron equivalent doses in several organs evaluated by the different works already discussed. From these data, it is possible to extract the ranges of neutron equivalent doses that a child could receive as a consequence of the brain treatment. In **Figure 1**, the effect of patient age can be noticed. For the thyroid, a close organ to the target, equivalent doses are similar among the phantoms, with an average of 1.69 mSv/Gy (RBE). In the rest of the organs, older patients do present lower doses. Equivalent dose in lungs ranges from 1.37 mSv/Gy (RBE) in the 1-year-old phantom to 0.83 mSv/Gy (RBE) in the 15-year-old phantom. In the abdomen, for instance, liver equivalent doses range from 0.93 mSv/Gy (RBE) to 0.49 mSv/Gy (RBE) for the 1-year-old and 15-year-old phantoms, respectively. Equivalent doses are below 0.5 mSv/Gy (RBE) in bladder for all phantoms. Equivalent doses in breasts have higher doses for all the phantoms [between 3.16 and 1.75 mSv/Gy (RBE)]. This is a consequence of the shallow depth of the tissue, which makes it more exposed to external neutrons. Consistently, male phantoms had a higher equivalent dose in comparison with female phantoms.

**Figure 2** compares equivalent doses for two different PS-PBT facilities. The phantoms considered were both 5 years old and with a similar target volume. Although simulation was done for a male phantom in Matsumoto et al. (31) while Sayah et al. (34) irradiated a female phantom, differences in sizes and organ configuration could be considered negligible for 5 years. Matsumoto et al. (31) found doses 3 times higher on average, except for breast, which can be explained by the fact that they used just one lateral incidence in comparison with the 5 incidences of the treatment in Sayah et al. (34). Matsumoto et al. (31) reported that previous measurements in the facility had shown higher ambient dose equivalent in the room in comparison to other facilities. In any case, all these facts agree with higher doses. The contradictory result in breast could be a consequence of reporting the dose as an average over both breasts considered as an organ. For one lateral incidence, the closest breast to the nozzle will be significantly more exposed than the contralateral breast, while with 5 field incidences, both breasts are more homogeneously exposed.

**Figure 3** shows results from Zacharatou-Jarlskog et al. (33) for the same facility and the same phantom. Treatment 1 (T1) had a target volume approximately 5 times larger than treatment 2 (T2) where one expected lower doses for the latter. However, except for breasts, results showed an opposite behavior. While T1 consists of a lateral field with a proton energy of 164 MeV, T2 consists of an oblique-inferior field of 180 MeV protons. It can be inferred from this result that the proton energy is the dominant parameter for neutron equivalent dose, which can even offset the effect of beam incidence and target volume. The behavior in breast is also related to beam incidence and breast position in phantom. For this irradiation, neutron equivalent doses in thyroid were 1.6 and 1.9 mSv/Gy (RBE) for treatment 1 and treatment 2, respectively. These values were similar to those reported by Sayah et al. (34), even in esophagus. For the other organs, a combination of incidence and specific configuration of beam lines could explain the differences.

Based on the works reviewed, we could establish that in PS-PBT facilities, a brain treatment could represent a neutron equivalent dose between 1.6 and 6.4 mSv/Gy (RBE) in thyroid, 4.1 and 0.51 mSv/Gy (RBE) in lungs, 2.6 and 0.12 mSv/Gy (RBE) in stomach, and below 0.1 mSv/Gy (RBE) in bladder. Assuming a prescription of 54 Gy (RBE) (30, 34, 36), total equivalent doses associated to the whole brain treatment would be approximately 216 mSv, 126 mSv, 73 mSv, and below 5.4 mSv for the thyroid, lungs, stomach and bladder, respectively.

Relatively scarcer works can be found for PBS-PBT facilities. In these facilities, while not using a significant number of absorbers in the beam line, the external neutron contribution from the treatment head becomes negligible (37). Ardenfors et al. (36) compared by MC simulation total absorbed doses in an adult and a 5-year-old patient irradiated by a pencil beam scanning system. They also considered a SUP and a LAT field. For this delivery system, neutron doses are essentially due to internal neutrons and therefore, the impact of field parameters could be different from those described in a passive facility. In fact, LAT field led to lower absorbed doses than with the SUP field, except in one of the eyes. Equivalent dose was reported in thyroid and bladder for the SUP field. The results were 62 µSv/Gy (RBE) and 2 µSv/Gy (RBE), respectively. These values represent approximately 39- and 50-times lower doses regarding PS-PBT.

Experimental studies in scanned-beam facilities have been done by EURADOS Working Group 9 (38, 39). In their campaigns, a 5-year-old and a 10-year-old anthropomorphic phantom were irradiated with 2 incidences (LAT and oblique) and several types of dosimeters located inside. They were able to evaluate the contribution of photons and neutrons. Knežević et al. (38) reported photon absorbed doses in organs and neutron dose equivalent as a function of distance to target center (only in the 5-year-old phantom). For example, in thyroid, they measured approximately 48 and 25 µGy/Gy (RBE) for the 5- and 10-year-old phantoms, respectively. In general, results in the younger phantom were approximately 2 times higher. For this phantom, photon doses ranged from 47 to 0.1 µSv/Gy (RBE) from 12 to 50 cm. In comparison, neutron dose equivalent ranged from approximately 200 µSv/Gy (RBE) to 3 µSv/Gy (RBE) from 12 to 36 cm. The results imply that neutron stray dose is still more relevant than photon dose in PBS-PBT.

**Figure 4:**
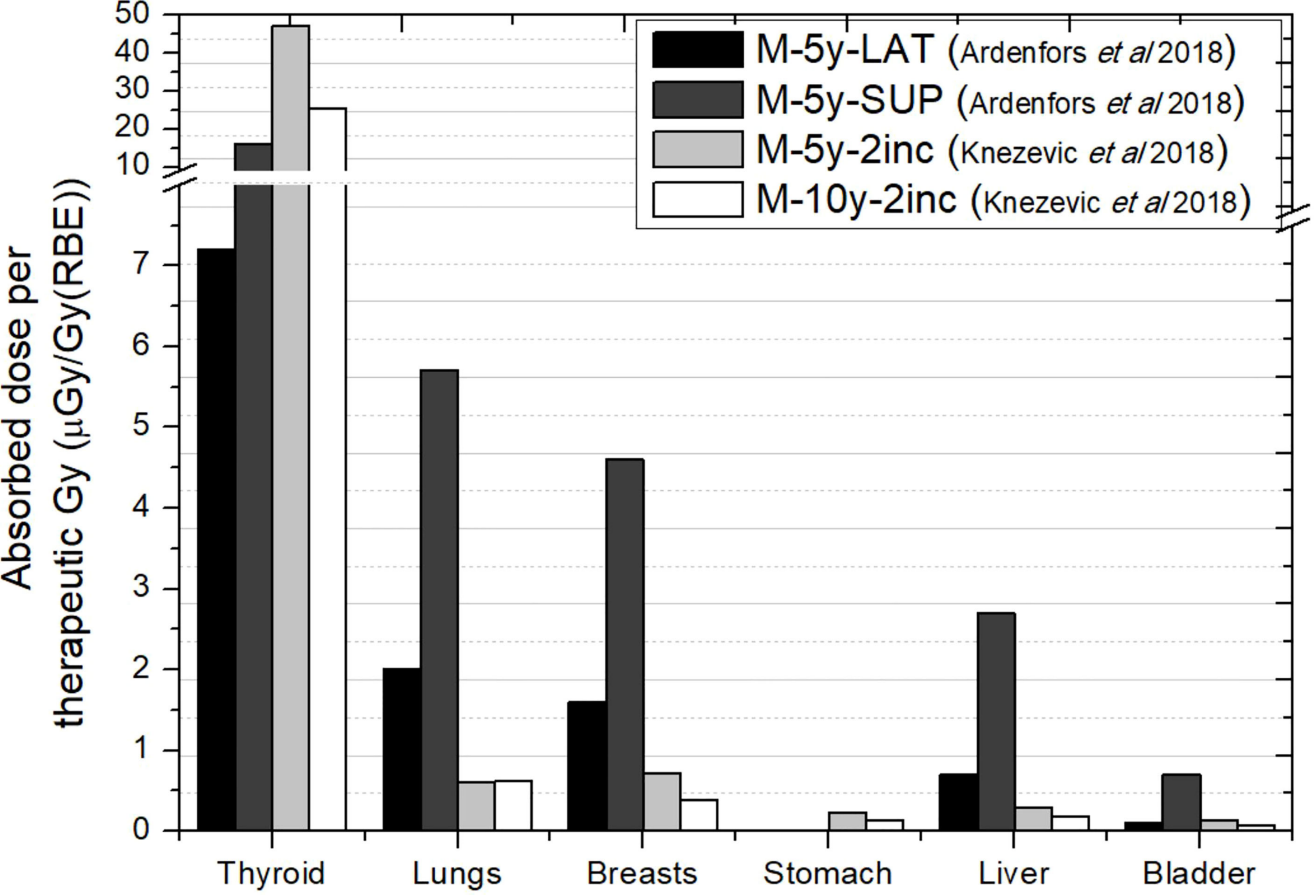
Absorbed doses per therapeutic Gy in selected organs for a brain treatment in two different proton pencil beam scanning facilities. Data extracted from Ardenfors et al. (36) (total absorbed dose) and Knežević et al. (38) (photon absorbed dose). In the legend, the sex (F, female; M, male), age of the patient (#y=number of years) and number of incidences (#inc = number of incidences) are specified. LAT, Lateral field; SUP, Anterior–superior field.

**Figure 4** shows the comparison between Ardenfors et al.’s (36) total absorbed doses and photon absorbed doses in Knežević et al. (38). As can be noticed, results are not compatible in thyroid, taking into account that Ardenfors et al. (36) results included both photons and neutrons. Some explanation can be found in the different target volume and the proton energy range of treatments in both works. While, in Knežević et al. (38), the target has a volume of 65 cm^3^ and proton energies range from 70 to 140 MeV, in Ardenfors et al. (36), these parameters were 24 cm^3^ and 80–110 MeV in the lateral field and 92–124 MeV in the superior field. Therefore, higher values were expected in Knežević et al. (38), although there must be other facts behind the observed inconsistency. For the remaining organs, results in Ardenfors et al. (36) were on average 5 times higher than in Knežević et al. (38). Given the result in thyroid, it is not possible to assign this difference only to neutron contribution.

In Wochnik et al. (39), the irradiation was repeated maintaining the target volume but shallowly located due to the use of an RS or a 3D printed beam compensator (BC). The introduction of these elements in the proton beam becomes a source of external neutrons and, therefore, a behavior in between PS-PBT and PBS-PBT would be expected. In this campaign, they reported both photon and neutron equivalent doses in selected organs for the 10-year-old phantom. They found, in general, a worse scenario with the RS despite of being farther from the patient. For this worst case, total equivalent doses ranged from 1.5 mSv/Gy (RBE) in thyroid to 39 µSv/Gy (RBE) in the bladder. Interestingly, equivalent dose in thyroid becomes similar to the values reported in passive scattering facilities. However, there is a faster reduction as the distance to target increases.

More studies for PBS-PBT would therefore be needed in order to be able to establish a range of equivalent doses received by the young patients.

### Out-of-Field Doses in Craniospinal Irradiation

In brain treatments, the beam points to the superior edge of the patient, and CSI covers the brain and the whole spinal cord. Therefore, the target presents a significant increase in terms of volume. In addition, as the spinal fields cover almost the whole trunk of the patient, organ distance to target will be lower than in the brain irradiation. Another important difference is that an almost whole-body CT scan of the patient is available and, therefore, can be included in simulations instead of using a voxel phantom. This is the case for the studies discussed in this section and, in addition, unless otherwise indicated, all modeled passive facilities.

**Figure 5:**
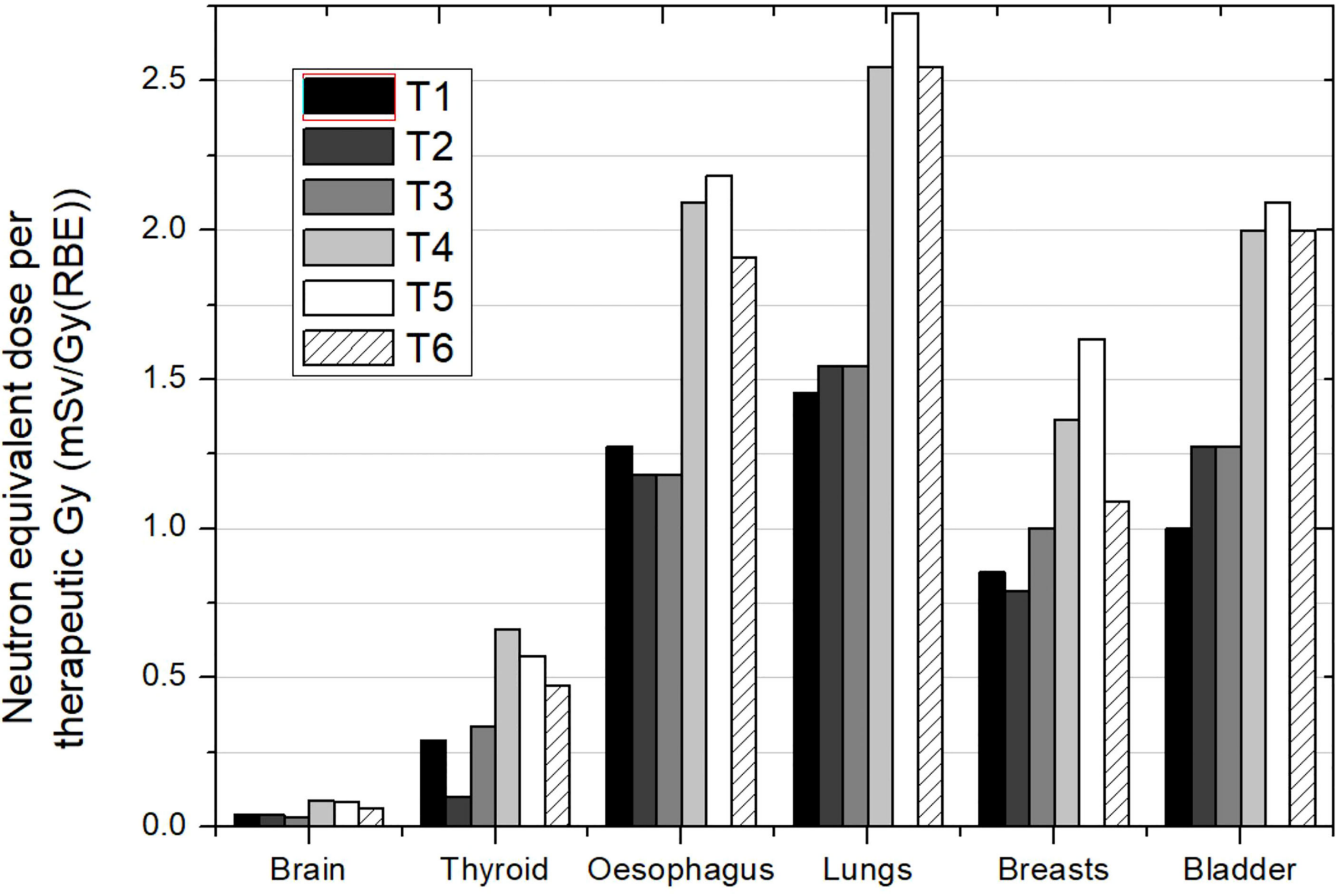
Neutron equivalent doses per therapeutic Gy in selected organs for the 6 different spinal fields in the same passive scattering proton beam facility. T1 (aperture diameter = 3 cm), T2 (aperture diameter = 6 cm), and T3 (aperture diameter = 9 cm) had a range and a modulation width of 10 and 5 cm, respectively. T4 (aperture diameter = 3 cm), T5 (aperture diameter = 6 cm) and T6 (aperture diameter = 9 cm) had a range and a modulation width of 15 and 10 cm, respectively. Data extracted from Athar and Paganetti (37).

Behaviors discussed in the previous section in relation to the influence of field parameters, such as beam incidence, range, modulation, or field size, are expected to occur in this case. For example, Athar and Paganetti (37) performed a simple estimation of the neutron equivalent doses expected for spinal fields in a passive facility. The aim was to compare the same fields used by Zacharatou-Jarlskog et al. (33) but with the proton beam directed posterior to the lumbar spine. **Figure 5** shows the neutron equivalent doses in several organs for the 6 fields considered (see details in the figure caption). As expected, organs in the trunk have now higher doses than in the head and neck region. Another expected behavior, discussed in the previous section, is that equivalent doses for the fields with smaller range and modulation width (T1, T2, and T3) are substantially lower than doses in the other fields (T4, T5, and T6). Their results showed that the maximum dose was 4.5 mSv/Gy (RBE).

Contributions from Taddei and collaborators (40–42) and Zhang et al. (43) did consider realistic CSI treatments. Usually, this treatment consists of a first plan covering the brain and the spinal cord with an additional boost in the brain. Each irradiation has its own prescription dose, and then the comparison of the whole plan in terms on equivalent dose per therapeutic Gy becomes difficult. Therefore, the analysis will focus on the treatments without the boost. Some works even did not simulate the boost fields assuming that the boost volume is small and located far away from the organs, and boost fields may contribute very little to the equivalent dose (42). Zhang et al. (43) also presented the dose distribution of stray neutrons overlapping the CT of the patient, showing how neutrons penetrate the whole body.

**Figure 6:**
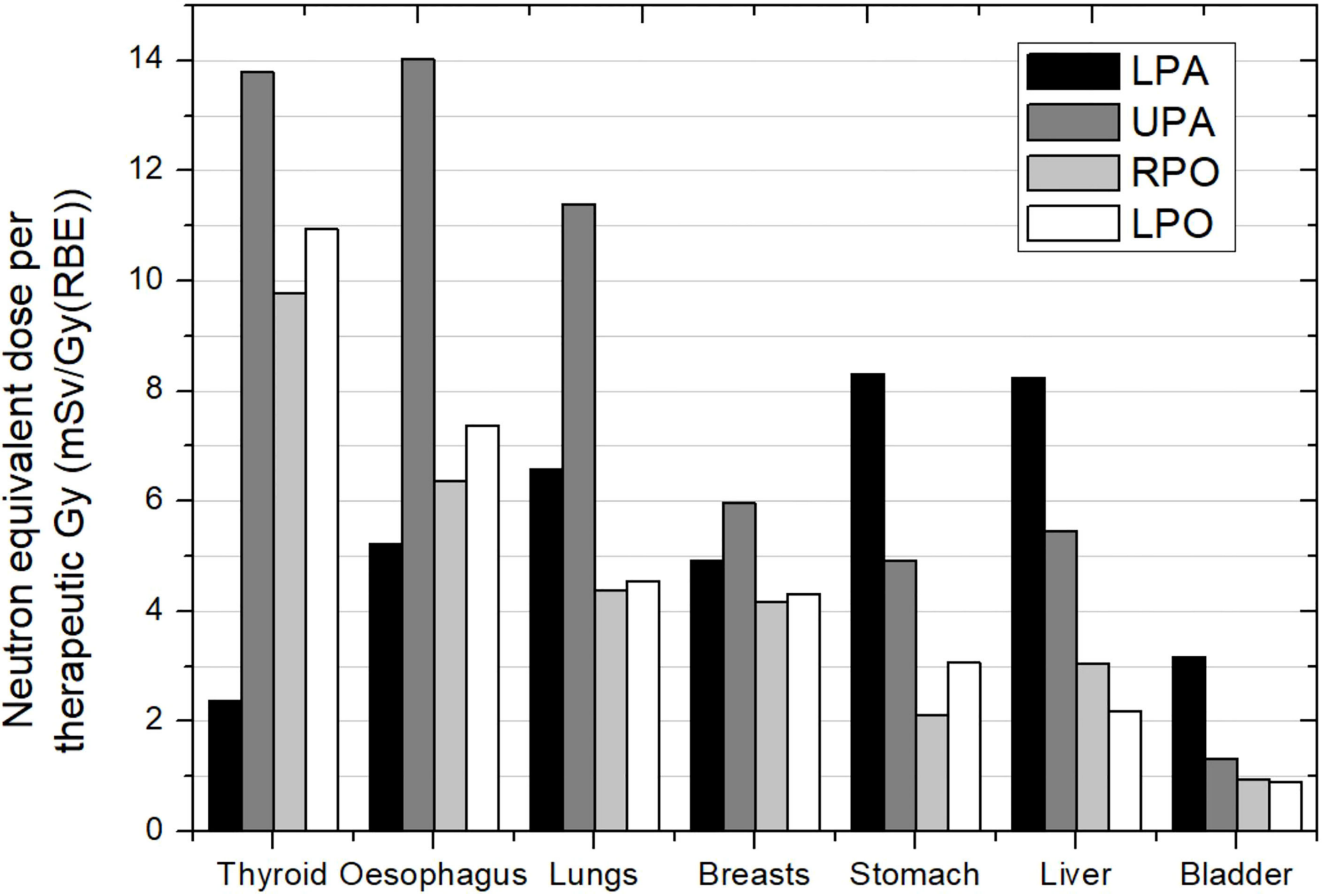
Neutron equivalent doses per therapeutic Gy in selected organs for the different incidences in CSI. Spinal fields: LPA, lower posterior anterior; UPA, upper posterior anterior. Cranial fields: RPO, right posterior oblique and LPO, left posterior oblique. Data extracted from Taddei et al. (40).

Taddei et al. (40) showed the different contribution of the incidences used for covering the target. In the case of a 10-year-old male patient, for a lower–posterior–anterior (LPA) field, the stomach, liver, and colon received the highest equivalent doses, approximately 8 mSv/Gy (RBE) each. For the upper–posterior–anterior (UPA) field, the esophagus, thyroid, and lungs received the highest equivalent doses, in the interval of 11 to 14 mSv/Gy (RBE). For cranial fields, bone surface and thyroid received the highest equivalent doses, each over 10 mSv/Gy (RBE). **Figure 6** shows equivalent doses in several organs for this case. The behavior of organ doses is related to the distance to the corresponding target. Taking as reference the organ order in the *x*-axis in the plot, we can see how equivalent dose decreases as moving to the right for the cranial fields. Meanwhile, the field directed to the upper part of the spinal cord leads to higher doses in the organs located in the thorax. Breasts present significantly lower doses than the others (thyroid, esophagus, and lungs), which can be easily explained by the opposite position in the body regarding the beam entrance. This means that caution is required when referring to distance, as distance in cranio-caudal direction, which can be representative in the majority of irradiations, for instance in brain, is no longer suitable in CSI.

**Figure 7:**
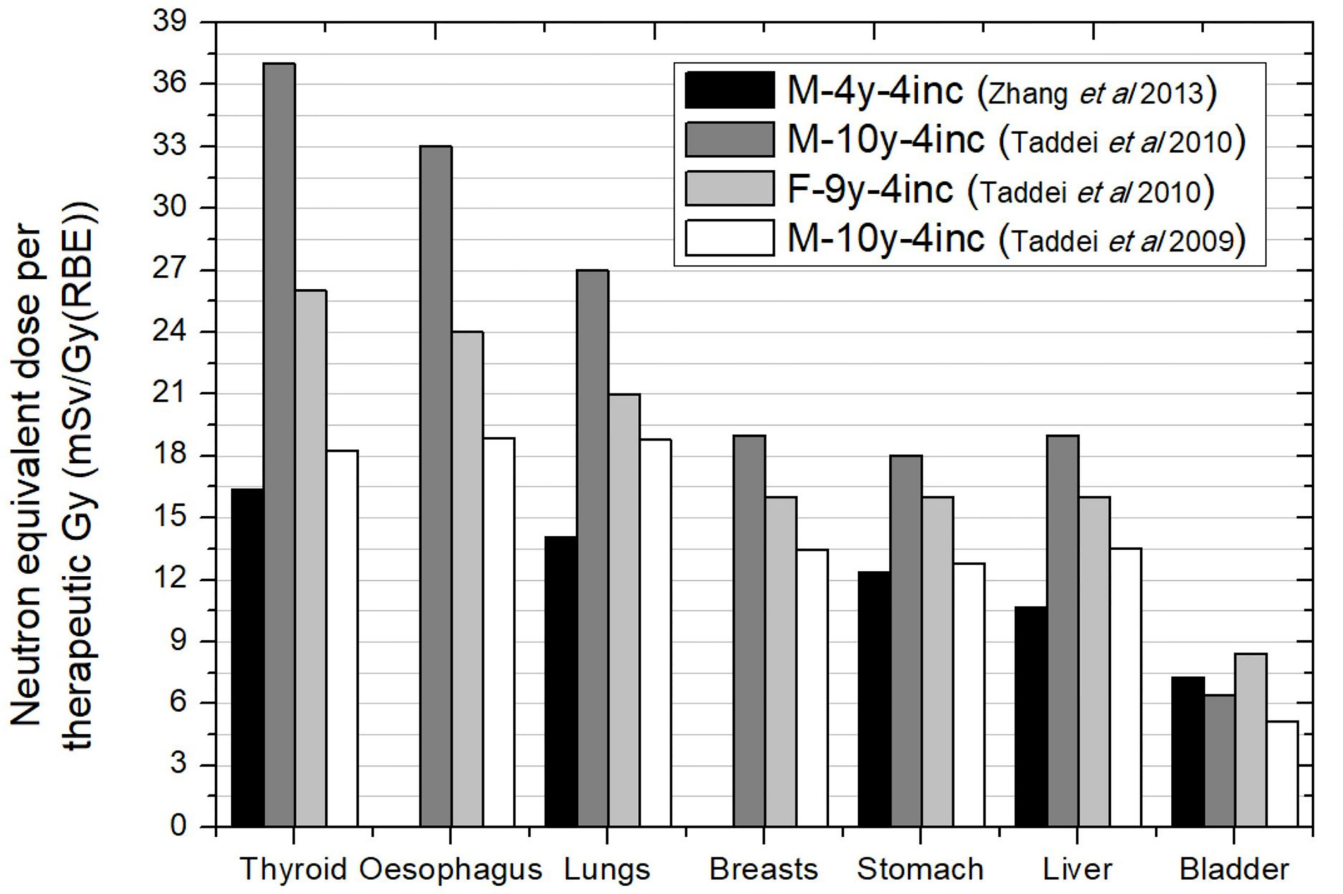
Neutron equivalent doses per therapeutic Gy in selected organs for CSI without boost for different passive scattering proton beam facility facilities. Data extracted from Taddei et al. (2009), (2010) (40, 42) and Zhang et al. (43). In the legend, the sex (F, female; M, male), age of the patient (#y = number of years) and number of incidences (#inc = number of incidences) are specified.

**Figure 7** shows the neutron equivalent dose for the whole treatment without the boost. Data were obtained for different patient ages. The first most striking aspect is that, in general, lower doses were found for the youngest patient. The effect of patient age in brain treatments is a consequence of the lower distances in younger patients. However, the large target in CSI makes distances in general similar between the children. Only for bladder can the age effect be slightly observed. All irradiations considered the same pattern of 4 beam incidences and, therefore, equivalent dose deviations may be attributable to differences in specific parameters of the incidence due to particular patient geometry. Based on the values reported, neutron equivalent doses range from 16 to 37 mSv/Gy (RBE) in thyroid, 14 to 27 mSv/Gy (RBE) in lungs, 12 to 18 mSv/Gy (RBE) in stomach, and 5.1 to 8.4 mSv/Gy (RBE) in bladder. These values represent an increase of one order of magnitude in comparison to brain irradiation illustrating the effect of the larger irradiated volume for CSI cases.

The worst scenario in **Figure 7** was for a 10-year-old male patient. If we add the contribution of boost fields (consisting in a left posterior oblique and a left lateral fields), we can evaluate total equivalent doses due to the whole treatment using the prescribed doses to the targets [23.4 Gy (RBE) in primary plan and 30.6 Gy (RBE) in the boost]. The results are that equivalent doses in CSI could reach values of 884 mSv, 715 mSv, 504 mSv, and 176 mSv in thyroid, lungs, stomach, and bladder, respectively.

These doses could be reduced with few modifications in the dimensions or material of some elements in the beam line as shown by Taddei et al. (41). They proposed increasing the thickness (from 4 to 8 cm) and changing the material (from brass to tungsten) of the field-defining collimator to improve the shielding in the nozzle. Additionally, a pair of jaws made of tungsten alloy were introduced in the nozzle to minimize the edge-scatter effects. This modification led to a percentage of reduction in equivalent dose from 33% to 59% in spinal fields and from 10% to 26% for the cranial fields.

Newhauser et al. (44) dealt with CSI in PBS-PBT. In fact, they compared the neutron equivalent doses in passive and scanned-beam facilities. Results from this work were not included in the previous discussion as they used results in adults to estimate the dose in a 3-year-old patient. Although the age of patient may not be so relevant as in brain irradiation, their assumption may bias the reported dose ranges. However, the comparison between passive and scanned-beam delivery system appears reasonable, especially when there is a lack of studies in scanned-beam facilities. Their results showed that the equivalent dose was on average two times lower in PBS-PBT.

**Figure 8:**
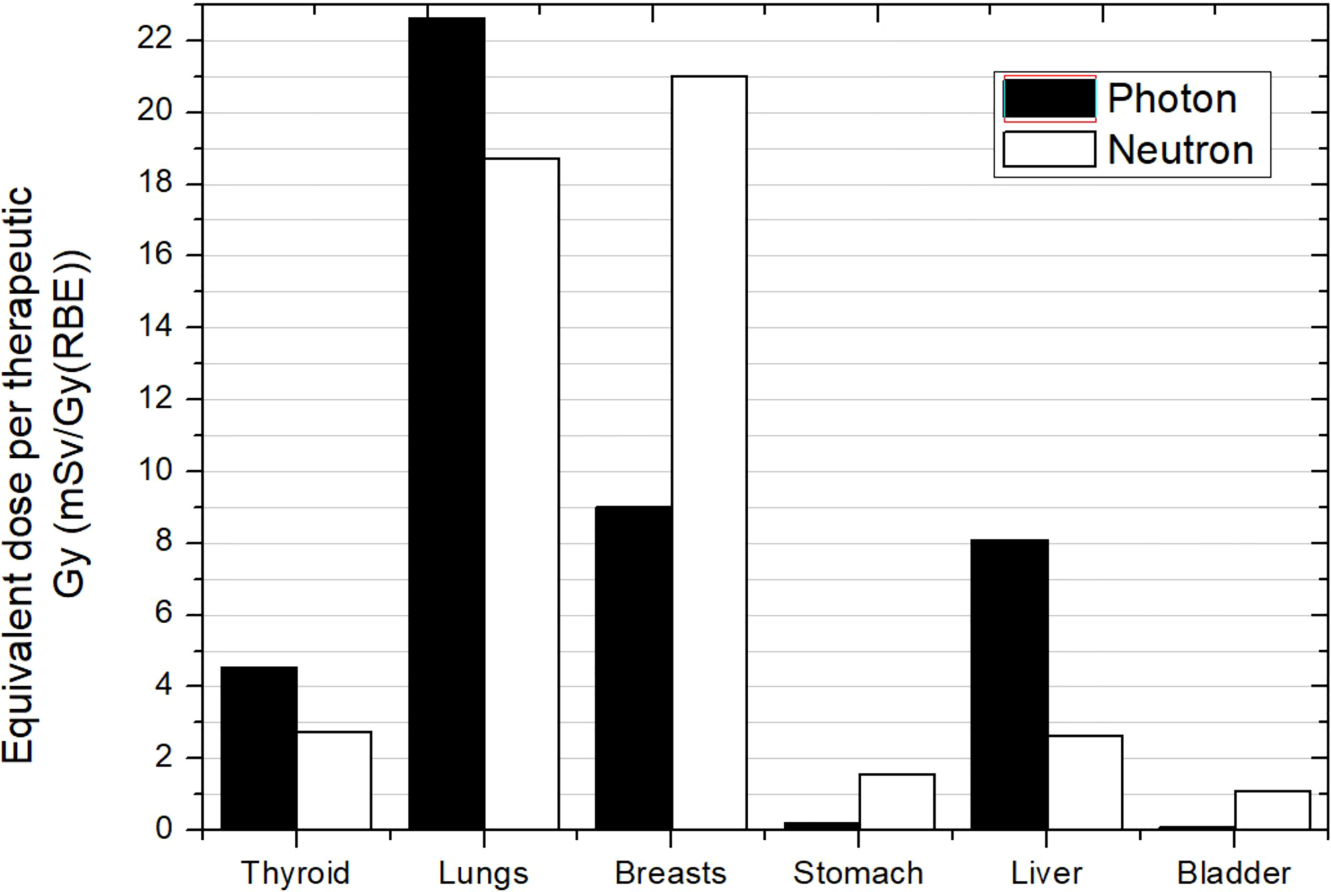
Photon and neutron equivalent doses per therapeutic Gy in selected organs for CSI in a proton pencil beam scanning facility for a 10-year-old anthropomorphic phantom. All fields included a range shifter. Data extracted from Majer et al. (45).

The work of Majer et al. (45) was the only experimental work and considered a PBS-PBT facility. They evaluated photon and neutron equivalent doses in selected positions inside a 10-year-old anthropomorphic phantom. The treatment consisted of two lateral fields for brain irradiation and three posterior anterior fields for spinal cord irradiation, all using an RS. Results for some organs are depicted in **Figure 8**. First, it can be noticed that a general trend cannot be derived when comparing photon and neutron contributions. While thyroid, lungs, and liver show higher photon equivalent doses, breasts, stomach, and bladder show lower photon doses. If we compare the neutron equivalent doses with data in **Figure 7** for PS-PBT, the most noteworthy result is that lungs and breasts are in the same range [approximately 20 mSv/Gy (RBE)]. This effect was also seen in brain irradiation when using the RS. However, neutron equivalent doses in other organs are lower in the pencil beam facility, by a factor between 5 and 10, higher than the factor 2 reported by Newhauser et al. (42). For example, the equivalent dose in thyroid was 2.7 mSv/Gy (RBE), while in PS-PBT, values can be up to 37 mS/Gy (RBE).

## Second Cancer Risk Estimates

The estimation of second cancer risk is in general subject to considerable uncertainty and often affected by the choice of model (46). As a consequence, absolute values of risks should be taken with caution, although relative comparisons can be regarded as more reliable, for example, using the ratio of risk values as a figure of merit (43). Lifetime attributable risk (LAR), i.e., the risk for the rest of life since treatment, is the usual endpoint for evaluating the probability of acquiring a second cancer due to the RT exposure. LAR for organs of interest is calculated using an appropriate model and then summed to obtain a global value.

Estimations of risks associated to neutrons were made by Zacharatou-Jarlskog and Paganetti (47). They simulated a passive scattered facility where voxel phantoms of different age were irradiated for a brain treatment. Using the formalism of BEIR-VII report (11), LAR was estimated assuming a treatment of 77 Gy (RBE). For calculation of LAR, a latency period and an expected maximum age attained must be considered. The BEIR report assumes a latency period of 5 years and a maximum age of 100 years. Phantoms covered ages from 9 months to 14 years and both sexes. Main results were significantly higher LAR values for female patients (about a factor of 2.5) and a fast decrease with age of exposure, especially at young ages. The fact that female patients present a higher risk is a direct consequence of the higher risk coefficients in the report (11), in accordance with epidemiological studies (48). The target volume also affected the risk, which became higher as the volume increases. As in their previous estimation of contribution of internal and external neutron to equivalent doses, they also established the contribution of each type of neutron to the risk. As expected, a similar trend was found: being the main component, the neutrons produced in the treatment nozzle between 82% and 98% of the total risk, depending on the beam parameters. Regarding the contribution of the different organs to total LAR, in male patients, solid cancers in lung and thyroid, together with leukemia, were of higher concern. For female patients, breast cancer is also included and even showing potentially the greatest concern. We have selected results from the treatment leading to the highest LAR for comparison with other works. **Tables 1**, **2** collect the LAR for the most radiosensitive organs and the total LAR for several of works discussed in the present section.

**Table 1:** LAR in thyroid and lung together with the total LAR for several irradiations in male patients at different ages.

Reference	Treatment	Patient age (years)	Therapeutic dose [Gy (RBE)]	LAR (%)		
				Total	Thyroid	Lung
Zacharatou-Jarlskog and Paganetti (44)	Brain	11	77	0.80	0.19	0.26
Geng et al. (49)	Brain	14	52.2	0.73	0.040	
Athar and Paganetti (46)	Brain	14	54	0.77	0.15	0.14
Zacharatou-Jarlskog and Paganetti (44)	Brain	14	77	0.55	0.15	0.15
Athar and Paganetti (35)	Spine	8	77	1.6	0.024	0.28
Athar and Paganetti (46)	Spine	14	54	1.4	0.020	0.33
Zhang et al. (41)	CSI	4	23.4	25		
Zhang et al. (48)	CSI	4	23.4	30	1	14
Taddei et al. (40)	CSI	10	54*	8.5	0.44	1.6
Zhang et al. (48)	CSI	15	23.4	8	0.2	4.0

*Including boost.

**Table 2:** LAR in thyroid, lung, and breast together with the total LAR for several irradiations in female patients at different ages.

Reference	Treatment	Patient age (years)	Therapeutic dose [Gy (RBE)]	LAR (%)			
				Total	Thyroid	Lung	Breast
Geng et al. (49)	Brain	4	54	4.5	0.5405		0.519
Zacharatou-Jarlskog and Paganetti (44)	Brain	4	77	5.5	2.2	2.4	5
Athar and Paganetti (46)	Brain	8	54	3.9	1.39	0.39	0.78
Athar and Paganetti (46)	Brain	8	54	0.35	0.18	0.04	0.04
Zacharatou-Jarlskog and Paganetti (44)	Brain	8	77	3	1.5	0.8	1.6
Athar and Paganetti (46)	Spine	8	54	4.2	0.18	1.16	0.68
Athar and Paganetti (46)	Spine	8	54	0.65	0.04	0.16	0.13
Athar and Paganetti (35)	Spine	11	77	3.2	0.221	1.05	0.708
Zhang et al. (48)	CSI	2	23.4	58	4	36	4
Taddei et al. (40)	CSI	9	54*	14.8	1.7	2.79	2.98
Zhang et al. (48)	CSI	16	23.4	11	1	5	2

*Including boost.

Athar and Paganetti (37) completed the previous work with results in spinal irradiations for a prescription of 77 Gy (RBE) as well. They found similar behaviors in terms of age dependence or target volume. For instance, LAR in younger patients was almost twice that for older patients. Spinal irradiation also led to a higher risk in lung and breast and, in addition, rectum for female patients and esophagus and rectum for male patients. In Athar and Paganetti (50), the prescription dose was reduced to 54 Gy (RBE) for similar brain and spinal irradiations. However, complete results were not provided for patients of the same sex and age, and therefore, a comprehensive comparison in terms of prescription dose cannot be done. It was only possible to compare LAR in the lung and breast for the 8-year-old female patient. Results were very similar, but it is not possible to ensure that the same treatment is compared and therefore any further conclusion cannot be easily drawn.

Total LAR is affected by the organs considered in the sum. Normally, if the most radiosensitive organs are considered, comparisons between LAR are still appropriate. However, the inclusion of skin or the remainder will significantly increase the total risk (51) and must be considered for meaningful comparisons. This is the case for Taddei et al. (42), for CSI irradiation, assuming a treatment with a prescribed dose of 23.4Gy (RBE) and a brain boost of 30.5 Gy (RBE), LAR values were 14.8% for a 9-year-old girl and 8.5% for a 10-year-old boy. LAR is reduced to 9.4% and 4.0% for the girl and the boy, respectively, when skin and the remainder are not considered. Their results agree with the fact that female patients have higher risks in general. For the CSI, the thyroid, lung, and breast were also major contributors to total risk.

Zhang et al. (43) evaluated the total dose received by the organs (not only due to secondary neutrons) to calculate LAR for CSI. Following also the BEIR report, they predicted a risk of 24.6% for an 8-year-old patient with a prescription dose of 23.4 Gy (RBE). If only stray neutrons had been considered, this risk would be reduced to 4.6%. Zhang et al. (43) considered the remainder for the total LAR but not the skin. If we subtract only the contribution of the skin to the value reported by Taddei et al. (42), we get a value of 6.2%, which agrees with the 4.6% reported by Zhang et al. (43). Nevertheless, the most important conclusion that can be extracted from the risk due to neutrons in comparison with total risk is that the contribution of neutron exposure comprises a much smaller proportion of the total risk (44).

The same methodology was followed in a retrospective study of 17 patients selected to be representative of a general population of children receiving CSI (52). The maximum and minimum values obtained for male and female patients are included in **Tables 1**, **2**. The LAR reported was up to 58% and 30% for a 2-year-old female and a 4-year-old male patient, respectively. Lungs were the organs with at significative higher risk in comparison with the others.

The BEIR report assumes the linear no-threshold model for risk estimates, which is appropriate for radiation protection purposes and for the low-dose region in RT (<4 Gy) (49). Stray neutron equivalent doses are maintained in the low-dose range; however, care must be taken when considering the region closer to the target, the so-called medium-dose region. For this range of doses, an appropriate risk model should incorporate the induction of DNA mutations, cell survival, cell repair, and repopulation, which occur during fractionated exposures as applied in RT. The model of Schneider based on the concept of organ equivalent dose (OED) and considering fractionation effects has been used as an alternative for risk estimation (53, 54). For example, Geng et al. (55) used the Schneider model for risk estimates in the brain tissue outside the target, resulting in a LAR approximately 0.5% and 3.2% for a 14-year-old male and a 4-year-old female patient, respectively. For the rest of the organs, the BEIR model was used for LAR calculation (some values are represented in **Tables 1**, **2**). If we sum the LAR in the non-target brain to the LAR in the rest of the organs, the total risk would be 0.73% and 4.5%, respectively. These figures are consistent with the behavior observed in the sense that a higher value is obtained for the younger and female patient. A limitation of these values is that they did not consider the lung, which has shown to have a high risk. Therefore, this risk estimate should be increased. Unfortunately, as they considered a brain treatment, it is not possible to directly compare their results with those from Zhang and colleagues (52).

Few works evaluated the risk for PBS-PBT (46, 50, 55). Their results showed an expected reduction in risk as a consequence of the reduction in stray radiation. For example, in Athar and Paganetti (50), total risks associated with stray neutrons were, on average, 10 times higher in passive beam in comparison to scanned beam. The average factor was approximately 4.7 when evaluating the whole stray radiation in the patient (55). In addition, Geng et al. (55) compared the effect of adding a patient-specific aperture. Their results showed that the risk increased, on average, by approximately 10% when adding the aperture. Moreover, there was a trend of increasing risk in organs as the distance from the target increases.

## Comparison With Photon Irradiation

Athar et al. (56) compared the results from Zacharatou-Jarlskog et al. (33) and Athar and Paganetti (37) for brain and CSI treatments to a 6 MV IMRT plan. They found similarities in the behavior of patient scatter and treatment head contribution when increasing the target volume in both modalities. However, while, for proton therapy, the secondary doses decrease with increasing distance to the field edge, IMRT fields show a rise in the absorbed photon doses at large distances due to accelerator head leakage. Consequently, close to the field, organs receive higher secondary neutron equivalent doses from PS-PBT relative to the scattered photon or leakage photon dose in IMRT. Conversely, organs located at larger distances from the field edge receive higher doses in IMRT than those in PS-PBT. They concluded that out-of-field doses from proton treatments seemed to be comparable to scattered doses received from 6 MV IMRT fields. As protons offer a distinct advantage in-field, proton therapy would represent a better option for children. However, as discussed in the previous section, these evaluated irradiations did not represent realistic treatments, at least for spinal fields. Therefore, disagreement could be expected with other works. In fact, Zhang et al. (43) also compared the CSI proton plan with a 6 MV IMRT. They found that for each organ, the equivalent dose was at least 1.5 times higher for photon therapy than for proton therapy. In fact, differences increased in thyroid, bladder, and colon, being a factor up to 28, 18, and 15, respectively. Majer et al. (45) compared a PBS-PBT CSI with 3D conformal RT and VMAT treatments. As photon treatments were planned with energies lower than 10 MeV, the contributions of neutrons could be neglected, and stray photons are the only contributors to out-of-field doses. Their results showed that, in general, total out-of-field equivalent dose is approximately 1 or 2 orders of magnitude lower for PBS-PBT compared to photon techniques. For lungs and breasts, 3D-CRT and PBS-PBT were comparable.

Comparison between proton and photon treatment can also be found in terms of risk. Athar and Paganetti (50) showed that PS-PBT offers an advantage for organs distant to the target, while closer to the field, the risk due to scattered dose in IMRT seems to be lower. These results were in agreement with those found for equivalent doses. However, comparison of total risk does lead to reduced values in proton therapy. Zhang et al. (52) showed that the CSI using a passive proton beam could reduce the predicted risk by 6 times. Brodin et al. (46) compared the risk in 3D conformal RT, rotational IMRT, and spot-scanned intensity-modulated proton therapy techniques for 10 patients receiving CSI for medulloblastoma. Their results showed that the scanned-beam proton therapy could reduce the risk by a factor of 7 in comparison to the photon techniques.

## Analytical Models

As described in previous sections, there are more MC studies in comparison to experimental ones. Some authors have referred to MC simulation as the gold standard. However, one of the disadvantages of simulation is the required time for calculations. Acute models of the geometry may require the use of computational clusters, and even the most simplified model could need several hours in a normal computer. This is the reason for the development over the last decade of several analytical models that allow a comparably quicker way for out-of-field dose calculation. These models enables both *a priori* and *a posteriori* estimations of dose. The former is useful in order to include the out-of-field information in the evaluation of a treatment. The latter is required for dose reconstruction in patient cohorts for epidemiological studies. A relevant contribution to this topic has been performed by Newhauser and co-workers mainly from Texas MD Anderson Cancer Center and Louisiana State University for PS-PBT, based on a detailed MC simulation of the nozzle, the cyclotron, and the treatment bunker (including wall, ceiling, and floor) (28). For brevity, some authors have referred to all this contribution as the LSU-MDA model. In a recent review, Newhauser et al. (28) performed a revision of these works and other available analytical models for photon and proton therapy. Since then, new extensions of the LSU-MDA model have been performed and tested in children. Gallagher and Taddei (57) adjusted the model to a clinical setting considering field parameters such as aperture size, range modulation, air gap between the treatment unit and patient, and radiation weighting factor. The model was applied to intracranial treatments and tested in two pediatric patients. The adjustment led to neutron dose equivalent estimates within a factor of 2 with the MC result. The Barandan et al. (58) model calculated in- and out-of-field neutron spectra and dose equivalent for pediatric CSI using a passive scattered proton beam, a Mevion S250 system. A double-Gaussian model of equivalent dose per proton absorbed dose using a fitted empirical parameter that apportions the relative dose contributions from high-energy and fast neutrons under reference conditions. Correction parameters related to brass aperture opening, modulation width, and thickness of the range compensator were incorporated. These models mainly consider the external neutrons that are the relevant in PS-PBT. However, PBS-PBT therapy has become the most common treatment in the last few years. Thus, one of the contributions of ANDANTE project (59) was a full parameterization of neutron absorbed dose, dose equivalent, energy quality factors, and RBE by Schneider and collaborators (60, 61). The parameterization was initially done for the Gantry 1 at the Paul Scherrer Institute (PSI), but it can be adapted to any other PBS-PBT facility. Neutron dose was modeled relative to the central axis dose, and three physical processes were considered for its description: dose build-up, inverse-square law, and exponential attenuation in a water phantom. They computed dose equivalent kernels as a function of water equivalent range and radial distance from the central axis of a single pencil beam for the nominal energies used at PSI. Depending on the specific plan, the appropriate kernel is applied at the position of each individual applied proton pencil beam of the field. The model was tested for two pediatric patients treated in PSI, one ependymoma and a cranio-spinal irradiation. The root mean square error between MC simulation and the model was up to 19% and 20% for absorbed dose and dose equivalent, respectively (61). Yeom et al. (62) also introduced a dose calculation method for reconstruction of the out-of-field neutron dose of pediatric patients based on a set of dose voxel kernels generated by MC simulation of proton pencil beams onto a water phantom with a size covering the body size of most pediatric patients. For each beam of a real plan, the dose kernel is matched with the CT of the patient to fix the first voxel irradiated and then rotated according to the direction of the proton beam. The total neutron dose is obtained accumulating the dose map for each beam. The model was tested with intracranial irradiation and CSI cases showing relative differences for most organs less than 30%. The authors highlighted the good performance regarding time, 30 min for the plan with the highest number of proton beams (7725), and regardless of the limitations, for example, the use of water instead of patient tissue with its heterogeneities, consider the model a useful tool for retrospective dose calculations to support epidemiological studies and for implementation into clinical TPS. Finally, an empirical model using a double-Gaussian function that related the voxel’s internal neutron equivalent dose per proton dose as a function of the shortest distance to field edge was developed by Gallagher and Taddei (63). The model was trained and tested using two intracranial pediatric treatments previously simulated. Their results showed that the applicable region of the model is from 3 to 49 cm, being more accurate from 3 to 10 cm, and differences with MC simulations are between 7% and 13%. For higher distances, the model overestimates dose equivalent by a factor between 2 and 3.

## Conclusions

An overview of the studies carried out to evaluate out-of-field doses and second cancer risk in young patients in proton beam therapy has been presented. The most widespread treatments are brain irradiation and CSI. As majority of works focused on passive scattering proton therapy, there is room for research in scanned-beam facilities, and, in general, from an experimental point of view, given that scarce works performed measurements. Based on the overview, neutron equivalent doses can be in the range of up to 1 mSv/Gy (RBE) in brain irradiation and 10 mSv/Gy (RBE) in CSI for PS-PBT. Photon contribution is approximately 10% of these values. For PBS-PBT, lack of data only allows to estimate that neutron equivalent doses decrease by a factor between 2 and 50 compared to passive scattering ones. Predicted risks of second cancer are higher in CSI in comparison to brain treatment, and absolute values are highly dependent on the age and sex of the patient. A wide range of values has been reported from lower than 0.35% up to 58%. These absolute numbers must be taken with caution due to uncertainties in risk models, but they can also be used for comparison between treatment techniques. Results discussed confirmed that proton beam therapy, especially PBS-PBT, leads to lower second cancer risks. Finally, there are some available analytical models for young patients, which can be useful to estimate out-of-field doses for evaluation of plans or to reconstruct dose for epidemiological studies. Differences with MC simulations showed that the estimations could be from 7% up to 50%, depending on the model and region in the patient. Therefore, improvement could be achieved in further investigations.

## Author Contributions

MR-E and AD contributed to conception and design of the study. MR-E collected the data, performed the analysis, and wrote the first draft of the manuscript. All authors contributed to manuscript revision, read, and approved the submitted version.

## Funding

This project has received funding from Euratom’s research and innovation programme 2019-20 under grant agreement no. 945196.

## Conflict of Interest

The authors declare that the research was conducted in the absence of any commercial or financial relationships that could be construed as a potential conflict of interest.

## Publisher’s Note

All claims expressed in this article are solely those of the authors and do not necessarily represent those of their affiliated organizations, or those of the publisher, the editors and the reviewers. Any product that may be evaluated in this article, or claim that may be made by its manufacturer, is not guaranteed or endorsed by the publisher.
